# Tissue sparing, behavioral recovery, supraspinal axonal sparing/regeneration following sub-acute glial transplantation in a model of spinal cord contusion

**DOI:** 10.1186/1471-2202-14-106

**Published:** 2013-09-27

**Authors:** Helen R Barbour, Christine D Plant, Alan R Harvey, Giles W Plant

**Affiliations:** 1 Department of Neurosurgery, Stanford Partnership for Spinal Cord Injury and Repair, Stanford University, Lorry I Lokey Stem Cell Research Building, 265 Campus Drive, Stanford, CA 94305, USA; 2 Eileen Bond Spinal Research Center, University of Western Australia, Perth, Australia; 3 School of Anatomy Physiology and Human Biology, University of Western Australia, Perth, Australia

**Keywords:** Transplantation, Tissue sparing, Behavioral recovery, Spinal cord injury, Glia

## Abstract

**Background:**

It has been shown that olfactory ensheathing glia (OEG) and Schwann cell (SCs) transplantation are beneficial as cellular treatments for spinal cord injury (SCI), especially acute and sub-acute time points. In this study, we transplanted DsRED transduced adult OEG and SCs sub-acutely (14 days) following a T10 moderate spinal cord contusion injury in the rat. Behaviour was measured by open field (BBB) and horizontal ladder walking tests to ascertain improvements in locomotor function. Fluorogold staining was injected into the distal spinal cord to determine the extent of supraspinal and propriospinal axonal sparing/regeneration at 4 months post injection time point. The purpose of this study was to investigate if OEG and SCs cells injected sub acutely (14 days after injury) could: (i) improve behavioral outcomes, (ii) induce sparing/regeneration of propriospinal and supraspinal projections, and (iii) reduce tissue loss.

**Results:**

OEG and SCs transplanted rats showed significant increased locomotion when compared to control injury only in the open field tests (BBB). However, the ladder walk test did not show statistically significant differences between treatment and control groups. Fluorogold retrograde tracing showed a statistically significant increase in the number of supraspinal nuclei projecting into the distal spinal cord in both OEG and SCs transplanted rats. These included the raphe, reticular and vestibular systems. Further pairwise multiple comparison tests also showed a statistically significant increase in raphe projecting neurons in OEG transplanted rats when compared to SCs transplanted animals. Immunohistochemistry of spinal cord sections short term (2 weeks) and long term (4 months) showed differences in host glial activity, migration and proteoglycan deposits between the two cell types. Histochemical staining revealed that the volume of tissue remaining at the lesion site had increased in all OEG and SCs treated groups. Significant tissue sparing was observed at both time points following glial SCs transplantation. In addition, OEG transplants showed significantly decreased chondroitin proteoglycan synthesis in the lesion site, suggesting a more CNS tolerant graft.

**Conclusions:**

These results show that transplantation of OEG and SCs in a sub-acute phase can improve anatomical outcomes after a contusion injury to the spinal cord, by increasing the number of spared/regenerated supraspinal fibers, reducing cavitation and enhancing tissue integrity. This provides important information on the time window of glial transplantation for the repair of the spinal cord.

## Background

In models of spinal cord injury (SCI), transplantation of growth-permissive tissues and cells has been performed to provide trophic support for damaged neurons and to transform the inhibitory milieu of the lesion site; this allows axons to grow through the otherwise impenetrable glial scar or cystic cavity. Olfactory ensheathing glia (OEG) facilitate the growth of newly-generated olfactory sensory axons into the olfactory bulb in normal adults [1-4] and after olfactory nerve section [5,6]. These cells have the potential to facilitate repair after injury, and their potential to enhance tissue repair has been extensively tested in experimental SCI models [7-14]. Schwann cell (SCs) grafts also have the ability to promote axonal regeneration following peripheral and CNS injuries [14-18], and as such were used in this study to compare the outcomes achieved using OEG in sub-acute spinal cord injury model.

Standard methods for isolating adult rat and human SCs have been developed, which result in a homogenous population of cells with stable characteristics [19-22]. However, methods to both isolate the OEG and transplant the OEG vary considerably, as do the experimental results. OEG display considerable morphological plasticity *in vitro*, changing phenotype within hours after changes in culture conditions [23-26]. Despite this, consistent functional and anatomical improvements have been obtained using olfactory bulb-derived p75-purified OEG from adult inbred rats [8-11,13,27]; results with lamina propria-derived OEG are less consistent [28-30].

To initially identify transplanted OEG and SCs, immunohistochemistry using an antibody to low affinity nerve growth factor receptor (p75) was used. However this approach is problematic, because it cannot unequivocally distinguish between transplanted OEG and SCs from endogenous SCs, which may enter the spinal cord via dorsal root entry zones or blood vessels [21,31-33]. Detection of p75 can sometimes be unreliable, as its ligand-binding domain can be cleaved *in vivo* and *in vitro* by endogenous proteases [34,35]. For this reason, lentiviral *ex-vivo* pre-labeling [12,26,36] of OEG and SCs with DSRED-2 was also used in this study. This allowed the quantification of surviving grafted cells, the analysis of their distribution and influence on endogenous spinal cord cells and axons, and assessment of their impact on matrix deposition and the host repair process.

We hypothesized that transplants of adult OEG or SCs may differ in their ability to promote axonal sparing/regeneration [4,37] and that a delayed transplant at 14 days post injury would improve anatomical and functional outcomes following a spinal cord contusion. This experimental study is based on numerous years of research into both glial types in CNS injuries, including the spinal cord. This time point was chosen because: (i) it represents a realistic time window deliver this type of cellular therapy in a clinical trial. This time period also gives consideration for time needed to generate and purify sufficient autologous OEGs for transplantation into injured patients, stabilization surgery and an optimal time window for best outcomes [38], (ii) experimental data from previous animal studies indicate that delayed transplantation may be more beneficial for cell survival, integration and reduced immune mediated rejection [8,14,20,38-41], and (iii) after 15 days *in vivo* a significant scar forms that may inhibit cell integration and axonal regeneration [38]. In support of this time point, 14 days was the time point chosen for the recent oligodendrocyte precursor and activated macrophage clinical trials. It should also be noted that the term “sparing/regeneration” has been used in relation to the analysis of axonal growth within this manuscript; this is because as described previously in a contusion model study [8] using fluorogold, we cannot truly distinguish between spared and regenerated axons.

## Results

### Cell transplantation is associated with improved retention of tissue at the lesion site

**Figure 1:**
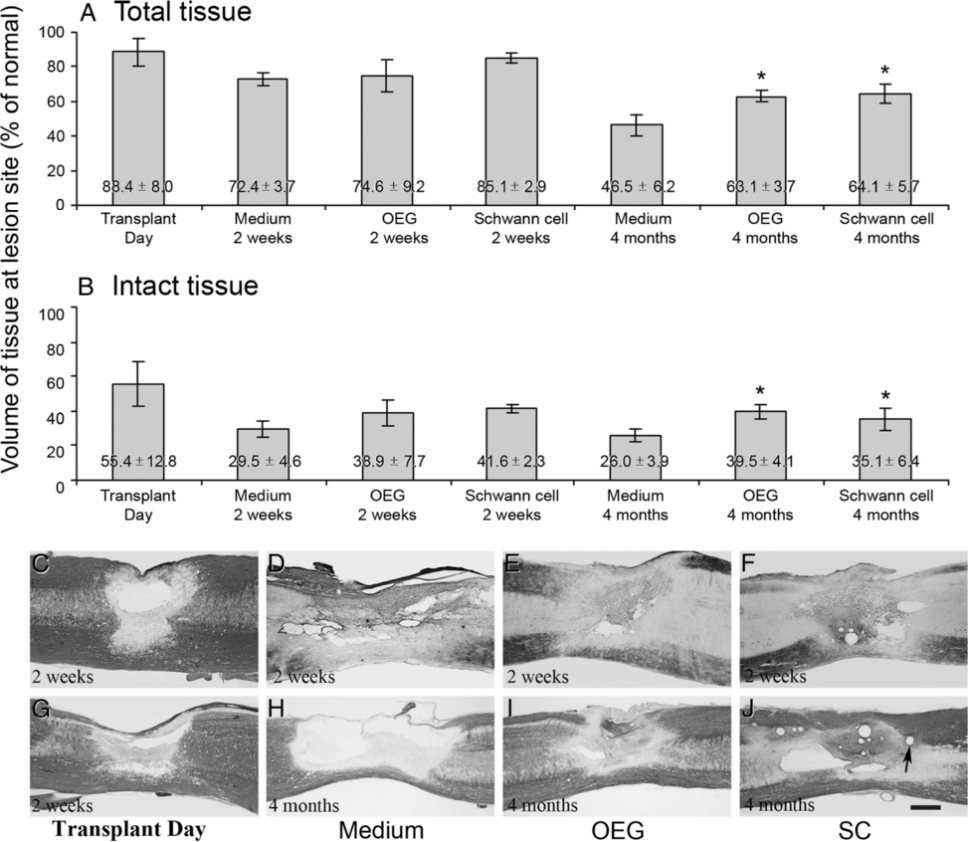
**Graphic describing the amount of total tissue remaining and intact tissue at the site of the spinal cord contusion injury at day of transplant 2 weeks and 4 months.** Quantification of gold chloride and Nissl stained sagittal sections to measure the amount of intact tissue and total tissue remaining within each of the experimental groups using Image Pro. **(A)**: Total tissue remaining 2 weeks and 4 months after cell transplantation. **(B)**: Intact tissue remaining 2 weeks and 4 months after cell transplantation. There was a significant increase (* p<0.05) in intact tissue found in the OEG and Schwann cell treated groups when compared to medium controls. **(C**, **G)**: Appearance of spinal cord lesion site 2 weeks after injury (i.e. day of cell transplantation) **(D)** Total tissue remaining at 2 weeks after medium injection. **(E)** Appearance of spinal cord lesion site 2 weeks after OEG transplantation (4 weeks after injury). **(F)** Appearance of the spinal cord lesion epicenter 2 weeks after Schwann cell transplantation (4 weeks after initial injury). **(H)**: Appearance of spinal cord lesion site 4 months after medium injection. **(I)** Demonstrates the spinal cord tissue 4 months after OEG transplantation. Note the considerable increased retention of intact tissue and a reduction in cystic volume when compared to image **(H)**. **(J)**: Injured spinal cord at 4 months after being injected with Schwann cells at the 14 day treatment time point. Note the presence of small microcysts (see black arrow) within the spinal cord parenchyma, but only where the Schwann cells are present. Scale bar: 200 μm.

All experimental groups exhibited loss of tissue at the lesion site following the initial contusion injury (Figure 1). Morphological analysis involved measuring the total amount of residual tissue; this included intact tissue, as well as the remaining graft and other degenerate/regenerate tissue that could not be classified as white or grey matter. The total tissue remaining included degenerate/regenerate tissue and the cellular trabeculae between cysts, lesion site and grafted cells. Tissue integrity classification was determined by Nissl gold myelin staining, which allows distinction between intact and non-intact tissue containing macrophages. The extent of the injury/transplant zone was also identified by the use of GFAP immunostaining, which allows the area of intact spinal cord versus the cyst areas to be defined. On the day of transplantation (i.e. 2 weeks post initial SCI), the total spinal cord tissue remaining was 88.4±8.0% of uninjured spinal cord and the amount of remaining intact tissue was 55.4±12.8% (mean±s.e.m).

Two weeks following cell SCs transplantation (OEG or SCs), there were no significant differences between groups in the amount of total tissue (p=0.27), with 72.4±3.7% >(n=6) of normal in the medium-injected group, 74.6±9.2% (n=6) in the OEG-transplanted group and 85.1±2.9% (n=6) in the SCs-transplanted group. Similarly, there were no significant differences (p=0.25) between groups for intact tissue remaining, which was 29.5±4.6% (n=6) of normal in the medium-injected group, 38.9±7.7% (n=6) in the OEG-transplanted group and 41.6±2.3% (n=6) in the SCs-transplanted group.

It was not until the 4 month time point that total tissue in the transplanted groups was found to be significantly greater than controls (p=0.028). Total tissue was 46.5±6.2% (n=11) of uninjured spinal cord tissue in the medium-injected group, 63.1±3.7% (n=11) in the OEG-transplanted group and 64.1±5.7% (n=11) in the SCs-transplanted group. A significant difference (p=0.028) was also seen between groups at 4 months in the amount of intact tissue remaining, which was 26.0±3.9% (n=11) of normal in the medium-injected group, 39.5±4.1% (n=11) in the OEG-transplanted group, and 35.1±6.4% in the SCs-transplanted group (Figure 1A, B).

### Nissl staining/gold chloride staining

Spinal cord sections visualized with Nissl staining showed good preservation of spinal tissue. Cysts that were present were frequently small (see Figure 1D,E and F) and very few in number. As mentioned above, no difference in tissue survival was noted in any of the control or transplanted groups (OEG or SCs). Nissl gold myelin staining at 4 months showed a reduction in tissue loss, particularly the reduction in cyst volume and sparing of white and grey matter within the lesion zone (see Figure 1H,I and J). The presence of round microcysts was noticeable in the spinal cords of SCs treated rats, and were present in 2 week and 4 month spinal cords; neither OEG transplanted rats or control (medium injected) rats showed these cysts.

### Transgene expression, cell dispersal and survival of transplanted Schwann cells and OEG

**Figure 2:**
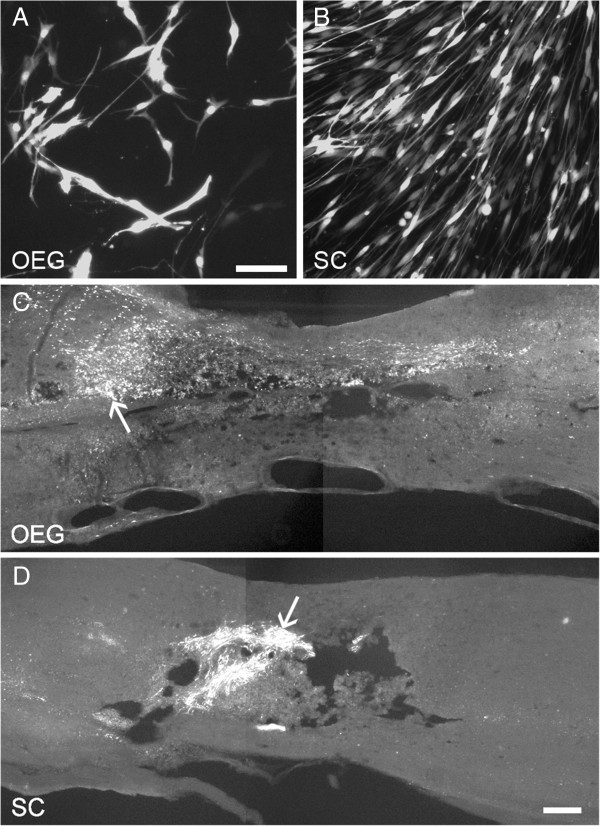
**Photomicrographs representing lentiviral transduced and transplanted OEG and Schwann cells. A**: OEG *in vitro* labeled with lentiviral DsRed2. **B**: Schwann cells *in vitro* labeled with lentiviral DsRed2. Scale bar **(A B)**: 50 μm. **C D**: Localization of transplanted OEG **(C)** and Schwann cells **(D)** within the spinal cord lesion site 2 weeks after transplantation. Schwann cells appear to have survived in greater numbers than the OEG. However, OEG are more dispersed through the spinal cord tissue while Schwann cells remain in a dense aggregate. Scale bar **(C D)**: 100 μm.

Approximately 92% of OEG and 99% of SCs were labeled *in vitro* (as a total of all Hoechst 33342 labeled cells) (Figure 2A and B). OEG or SCs were not previously transduced with self inactivating lentiviral-DSRED-2 vectors. This vector was found to be non-toxic (data not shown) and enabled visualization of cytoplasmic spread and cellular morphology for up to 4 months post-transplantation into the CNS. DSRED-2’s stability and capacity to track cells was determined to be as efficient as seen previously with GFP [12,26,36,42]. DSRED-2 labeled showed no additional immunological response *in vivo* when compared to the GFP transgene which may occur with LacZ [43] or human placental alkaline phosphatase [44].

Figures 2C and D show representative spinal cord sagittal sections with OEG and SCs grafts 2 weeks following cell transplantation. Lentiviral-DSRED-2 labeled OEG was detected in all but one of the animals whereas lentiviral-DSRED-2 labeled SCs was detected in all animals. At 2 weeks after transplantation to the injured spinal cord, DSRED-2 fluorescence pixel intensity for surviving transplanted OEG was 70±29 at the rostral aspects of the transplant/lesion zone, versus 91±37 for SCs, at the center of the lesion 86±35 for OEG and 100±41 for SCs. Lastly the DSRED-2 fluorescence pixel intensity at the caudal edge of the transplant/lesion was 83±34 for OEG and 79±32 for SCs. In rats at 2 weeks (n=6 per group) analyzed, detectable transplanted OEG had in some cases dispersed up to 5 mm from the center of the injection site, the furthest distance measured. In contrast, DSRED-2 positive transplanted SCs were highly localized to the site of injection, dispersing no more than 0.6 mm from the injection site. At 2 weeks post transplantation the surviving OEG were dispersed throughout the lesion site and adjacent spinal cord tissue, and were aligned along the rostro-caudal axis of the spinal cord. SCs were densely packed, with an alignment that was highly regular within the graft area but did not disperse into the intact host CNS tissue surrounding the lesion site.

Four months after OEG transplantation, DSRED-2 positive OEG were detected in 7 out of 11 animals (64%) whereas DSRED-2 positive SCs were detected in 11 out of 11 animals (100%). Measurements of DSRED-2 fluorescence pixel intensity showed OEG at the rostral part of the lesion at 22±9 and SCs 34±14, at the center of the lesion the values were 21±9 for OEG and 29±12 for SCs. DSRED-2 pixel fluorescence was found to be 19±8 for OEG at the caudal part of the lesion and 28±12 for SCs. As estimated from the DSRED-2 fluorescence, the levels of OEG present within the spinal cord had decreased throughout the rostral-caudal axis of the lesion by 74% at 4 months from the value seen at 2 weeks post transplantation. At the same time DSRED-2 fluorescence pixel values for SCs reduced from 2 weeks and 4 months, however fluorescence levels were significantly higher than OEG levels (p<0.05) (see Additional file 1: Table S1).

### Scar formation, CS56 synthesis and gliosis

**Figure 3:**
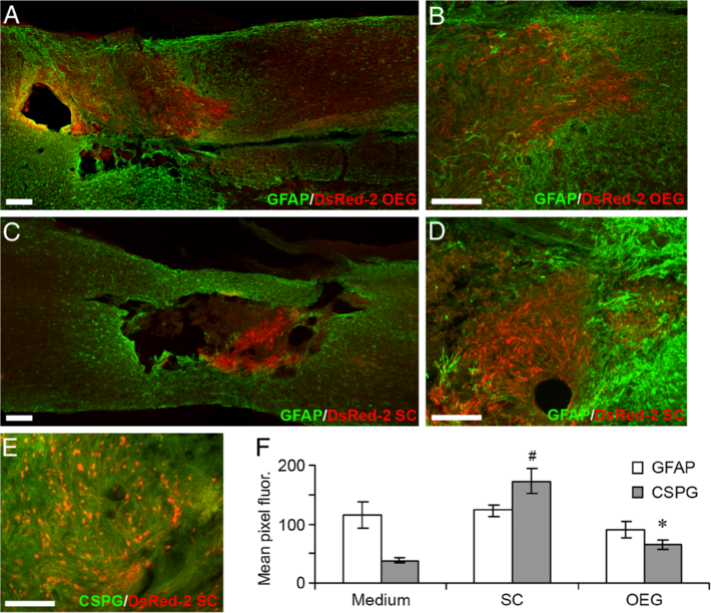
**Glial scar formation at the lesion site indicated by GFAP and CSPG immunoreactivity. A-D**: GFAP in green DsRed2-labeled transplanted cells in red. **A**-**B**: Lesion site 2 weeks after transplantation of OEG. **C**-**D**: Lesion site 2 weeks after transplantation of Schwann cells. Dispersal of transplanted cells is greater for OEG than for Schwann cells. Schwann cells remain in a dense aggregate at the lesion site surrounded by strong GFAP immunoreactivity. **E**: Schwann cells are surrounded by dense proteoglycan network CSPG (green), DsRed2-labeled transplanted cells (red). Scale bars: **A**-**D**: 100 μm, **E**: 200 μm. **F**: Graph showing the quantitative fluorescence (measured by mean pixels) of GFAP and CSPG at the distal interface of the transplant zone (n=10 for each group). OEG had significantly less (p<0.05) CSPG expression when compared to Schwann cells (see *). Schwann cells had significantly greater (p< 0.05) CSPG expression when compared to medium injected controls (see #).

Deposition of proteoglycan scar/inhibitory molecules was assessed in transplanted OEG, SCs, and control medium injected spinal cords by immunofluorescence section staining with antibodies to GFAP and CS56 (for chondroitin sulphate proteoglycans). The extent of staining was assessed across the lesion site in a rostro-caudal direction. After 2 weeks post transplantation, OEG spinal cords showed a moderate change in GFAP staining when compared to SCs and medium treated spinal cords (n=6 (Figure 3A,C). In particular, this was noted at the caudal aspect of the transplant zone (Figure 3B,D). At 4 months following OEG, SCs and medium injections, GFAP immunofluorescence staining (analyzed by pixel intensity) revealed a trend towards a lower degree of GFAP immunofluorescence at the injury zone. The OEG transplanted group showed a reduction of 27% in GFAP immunofluorescence when compared to the SCs transplanted group and a 23% reduction in GFAP immunofluorescence when compared to the medium-injected group. However, this difference was not statistically significant (p<0.05).

Integration of transplanted cells with host astrocytes was frequent for the OEG transplant groups but infrequent for the SCs transplant animals. CS56 proteoglycan staining was analyzed across the OEG, SCs and medium groups in the 4 month post transplant spinal cords (n=11). Immunofluorescence staining for CS56 in the SCs transplanted group (Figure 3E) was significantly greater than medium injected control sections (p<0.001) (Figure 3F) and the CS56 immunofluorescence was greater in the SCs group compared to the OEG transplanted group (p<0.01) (Figure 3F). CS56 immunofluorescence in the OEG group was not significantly different from the levels seen in the medium injected controls.

### Extracellular matrix deposition within the lesion site

**Figure 4:**
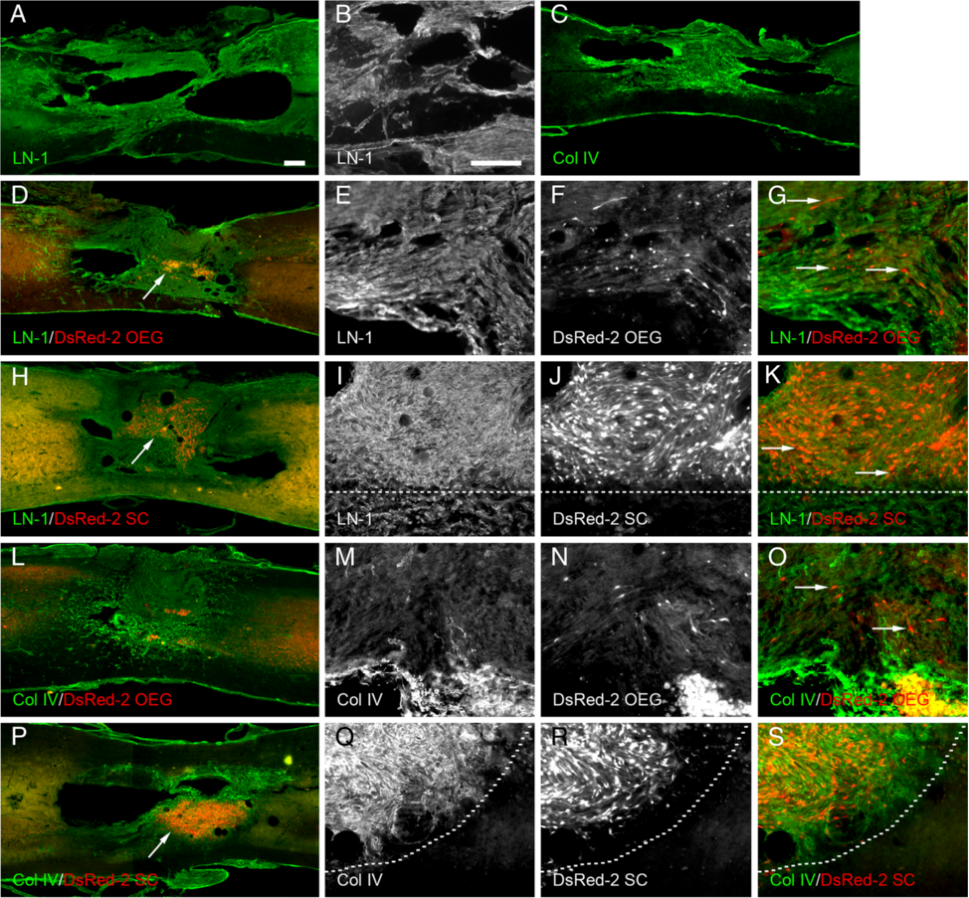
**Schwann cell, OEG and medium treated spinal cords at 4 months.** Photomicrographs depicting the distribution and immunoreactivity for laminin-1 **(A**, **B**, **D**-**K)** and collagen IV **(C**, **L**-**S)** at the site of spinal cord contusion lesion with or without the transplantation of SCs and OEG. Areas of strong immunoreactivity for laminin can be seen to surround the areas of the transplanted cells (see arrow in **D** depicting DsRed-2 OEG). Laminin immunostaining does not appear to be co-localized on the surface of the OEG cells **(**see arrows in **G)**. Laminin is also found surrounding Schwann cell transplants **(**see **H)**. Arrow depicts the central core of the transplant. Laminin is very strong in the region of the Schwann cells **(**see **I**-**J)** with the host spinal cord below the dashed line has reduced intensity of staining. Collagen IV immunostaining depicts a central core of staining close to the lesion site surrounding any surviving OEG **(**see **L)** but labeled cells** (**see arrows in **O)** are not co-labeled with collagen IV. Collagen IV immunostaining in Schwann cell transplanted spinal cords show intense staining surrounding and within the DsRed-2 SC transplant area (see arrow). In higher power the staining is mostly co-localized (see above dashed line) but some areas are not. Scale bars: 100 μm. Scale bar in A also serves **C**, **D**, **H**, **L** and **P**. Scale bar in B also serves **E**, **F**, **I**, **J**, **M**, **N**, **Q** and **R**.

At 4 months post injury, the grey and white matter bordering the lesion site contained numerous blood vessels, the walls of which were highlighted by laminin-1 staining (Figure 4A,B,D-K). The *dura mater* was strongly immunoreactive for laminin-1 in all analyzed spinal cords. In addition to the meningeal and vascular-associated laminin-1, the graft sites of OEG and SCs grafts also contained laminin-1. Compared to the medium injected control group, the laminin-1-stained area appeared more extensive in both the OEG and SCs transplanted groups, but these differences were not statistically significant (p=0.15). At higher magnification, it was observed that areas strongly immunoreactive for laminin-1 co-existed with the DSRED-2 positive cells only in the SCs transplanted group (see Figure 4I,J,K vs. E,F,G). Frequently, the areas of strong laminin-1 immunoreactivity in OEG grafted spinal cords were not associated with the location of surviving DSRED-2 positive transplanted cells, indicating another source or cell type such as endothelial cells for the laminin-1. Laminin-1-positive structures around bundles of axons in both the OEG and SCs transplanted groups were observed.

The profile of immunoreactivity for collagen IV (Figure 4C and L-S) was similar to that observed for laminin-1. Structures resembling blood vessels were visible around the site of the spinal cord lesion in the OEG transplanted group (Figure 4L). While the graft site of the OEG transplanted group contained more collagen IV immunofluorescence than that of the other groups (Figure 4L and P vs. C), measurement using pixel intensity revealed the collagen IV immunofluorescence for each group to be statistically similar (p=0.10). As with laminin-1, at higher magnification, collagen IV could be seen in high quantities in regions of the SCs grafts. However in the OEG transplanted group high amounts of collagen IV appeared to originate from DSRED-2 negative cells in the vicinity of the DSRED-2 positive cells (Figure 4O,S), which may represent either endogenous SCs invading from the periphery or host endothelial cells.

### Axonal profiles inside lesion site

**Figure 5:**
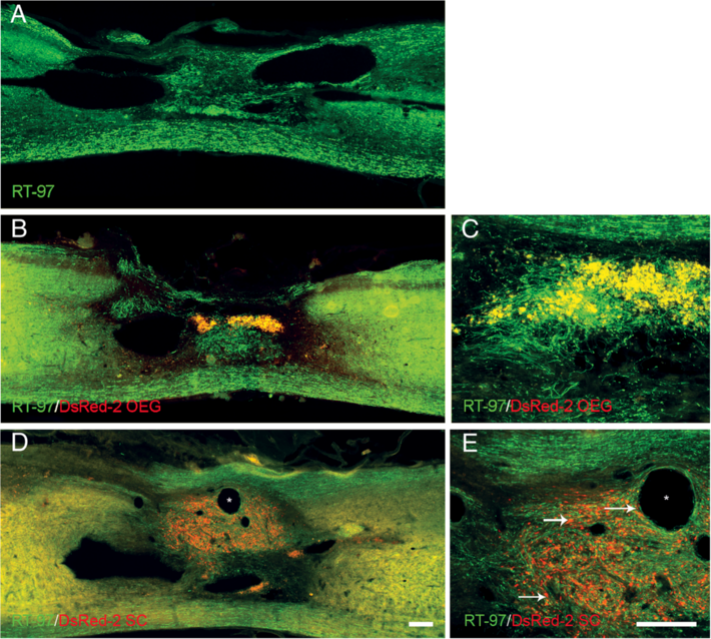
**Axonal staining at the lesion site of Schwann cell, OEG and medium groups.** Immunoreactivity for RT-97 at the site of spinal cord contusion 4 months after medium **(A)** OEG **(B**-**C)** and Schwann cells **(D**-**E)** transplantation. RT97 positive axons are observed within the lesion site in all groups but number and distribution varied. Axons in the OEG group **(B)** were arranged in fascicular bundles but did not have rostral to caudal orientation unlike axons beneath the graft area which constitute the ventral white matter. See **(C)** for higher power image of **(B)**. RT97 staining after Schwann cell transplantation **(**see **D)**. These axons showed a close association with DsRed-2 positive cells but followed a non linear path. Arrows in **(E)** depict DsRed-2 labeled Schwann cells deep within the transplants or close to microcysts (*). Scale bars: 100 μm. Scale bar in **D** also serves **A** and **B** scale bar in **E** also serves **C**.

Immunohistochemistry with the neurofilament-specific antibody RT-97 was used to estimate the presence of medium and high neurofilament-containing axons in and around the lesion site of all transplanted groups and controls (Figure 5). At the lesion epicenter of all groups, continuous white matter tracts of spared axons could be seen at the ventral edges of each section (Figure 5A,B, and D). Dorsal white matter tracts were in an improved state of preservation after OEG and SCs transplants. In addition, RT97^+^ axons were present in the lesion site in all transplanted and control groups with RT97^+^ axons being co-extensive with DSRED-2 OEG and SCs (Figure 5E). Axons entering SCs graft areas or putative graft areas, were commonly observed to lose rostral-caudal directionality and instead aligned with the random internal organization of the graft (Figure 5E). However this non-linear pathway for axons was not seen in the OEG transplanted spinal cords (Figure 5C).

### Evidence for infiltration of endogenous Schwann cells into the spinal cord

**Figure 6:**
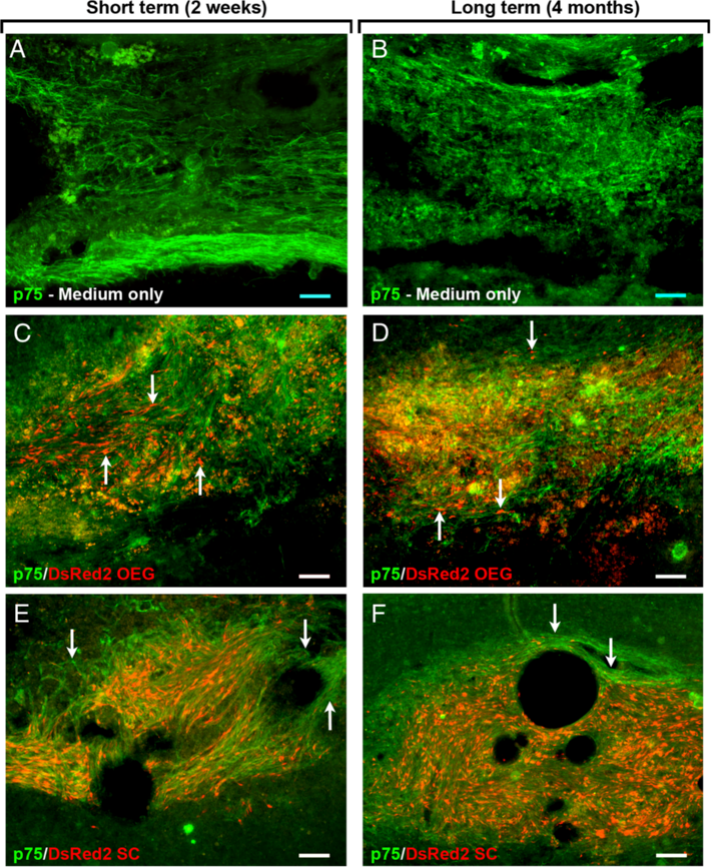
**Photomicrographs depicting p75 immunoreactivity of spinal cord sections in the lesion site after SCI.** Endogenous p75 positive cells Schwann cells are present within the lesion site 2 weeks **(A)** and 4 months post injury **(B)**. p75 positive profiles are present in large numbers in OEG **(C**-**D)** and Schwann cell **(E**-**F)** transplanted groups. The arrows in **C** and **D** represent DsRED-2 positive transplanted OEG within the lesion site co-mingled with host p75 positive Schwann cells. In the Schwann cell transplanted group the p75 positive endogenous Schwann cells (see arrows) also surround the transplanted DsRed-2 positive cells The Schwann cell transplants are tightly clustered unlike the dispersed OEG **(**see **C-D)**. Scale bar in **C** (100 μm) also serves **A** and **B**.

In several of the OEG or SCs transplanted animals, the lesion site contained numerous fascicles of cells with glial-like spindle-shaped morphology; these cells were positive for p75 but negative for DSRED-2. Sections from the medium-injected group also contained p75-positive cells both at 2 weeks and 4 months after transplant day (Figure 6A,B). The p75-positive cells in the OEG (Figure 6C and D) and SCs (Figure 6E,F) transplant groups may have been donor cells that were not expressing the DSRED-2 transgene. It is also possible that SCs had migrated into the spinal cord from blood vessels within the spinal cord or via the dorsal roots [45]. The latter is more likely, because the method using lentiviral vectors encoding transgene such as GFP have previously been shown to display long term expression [12] and these p75-positive cells were also detected in the medium-injected groups.

### Numbers of retrogradely labeled descending axons increased by glial transplantation

Retrograde labeling with Fluorogold was used to quantify extension of spared/regenerated descending supraspinal axons reaching 8 mm beyond the most caudal edge of the contusion site. Mean±standard errors of numbers of descending Fluorogold-labeled cells are presented. There were statistically significant differences (p<0.05) between the total brain counts of the OEG transplanted group (3019±216) and also the SCs transplanted group (2767±411) when compared to the control injury only group (1204±331).

**Figure 7:**
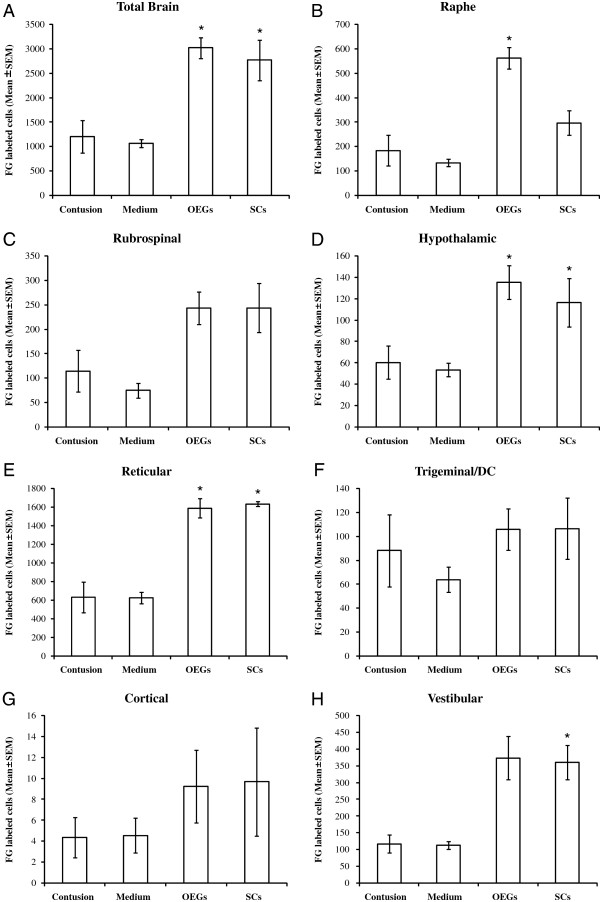
**Number of neurons in brain/brainstem nuclei retrogradely labeled with Fluorogold injected 8 mm caudal to the thoracic spinal cord lesion site.** OEG transplantation into the contused spinal cord 14 days after injury promotes significant axonal sparing/regeneration of Raphe-, Vestibular- and Reticulo-spinal axons. Graphs depict the mean numbers of total FG-labeled neurons in the brains and brainstems of the four groups **(A)**. Mean neuron numbers of the Raphe **(B)**, Rubrospinal **(C)**, Hypothalamic **(D)**, Reticular **(E)**, Trigeminal/DC **(F)**, Cortical layer V **(G)** and Vestibular **(H)**. Experimental groups were compared using a one-way analysis of variance (ANOVA), followed by the Dunnett’s method of multiple comparisons versus a single control group (injury only) and also the Tukey test for multiple comparison procedures between all experimental and control groups.

Statistical differences (p<0.05) were seen in a number of separate brain regions projecting distally through the injury zone. OEG transplanted rats showed statistically significant differences in the Raphe projecting neurons (562±43) compared to controls (184±62). Numbers of Fluorogold neurons in the Raphe for SCs transplanted rats (296±50) did not show significant differences when compared to control injuries only. Rubrospinal Fluorogold numbers in the OEG and SCs grafted groups were similar (243±34 and 244±50 respectively) and not statistically significantly different from the control injury only group (115±42). The numbers of Fluorogold labeled neurons in the hypothalamus were significantly higher in both the OEG and SCs groups (135±16 and 116±23 respectively) when compared to controls (60±15). Fluorogold labeled neurons in the reticular formation in control injury only rats numbered 632±165, as compared to 1590±100 for OEG treated rats and 1635±23 for SCs grafted animals (Table 1 and Figure 7). Fluorogold numbers in the trigeminal/DC were not statistically significantly different in all groups (Table 1 and Figure 7). This was also the case for corticospinal layer V neurons whose axons rarely seen to project into the distal spinal cord (8 mm) fluorogold injection site (Figure 7). Finally, neurons labeled in the vestibular system were significantly increased in the OEG and SCs transplanted rats (373±65 and 360±51 respectively) when compared to control injury only rats (117±27).

**Table 1:** Retrogradely Fluorogold-Labeled Neurons in Control, OEG Transplanted and SC Transplanted groups

	**Total brain**	**Reticular formation**	**Percent total**	**Hypothalamic**	**Percent total**	**Vestibular complex**	**Percent total**	**Raphe nuclei**	**Percent total**	**Rubrospinal**	**Percent total**	**Trigeminal/DC**	**Percent total**
Contusion only^*^	1204±331	632±165	53	60±15	5	117±27	10	184±62	15	115±42	10	88±30	7
*Medium only*^***#***^	1068±79	624±62	58	53±6	5	113±12	11	134±15	13	75±15	7	64±11	6
*OEG treatment*	3019±216^**#**^	1590±100^**#**^	53	135±16^**#**^	4	373±65^**#**^	12	562±43^**#**^	19	243±34	8	106±17	4
*SC treatment*	2767±411^**#**^	1635±23^**#**^	59	116±23^**#**^	4	360±51^**#**^	13	296±50	11	244±50	9	106±26	4

^*^The contusion only groups received a moderate contusion injury. Fourteen days after injury, OEG or Schwann cells were transplanted into the lesion site; controls received an injection of cell culture medium. Data are presented as means ± SEM.

^#^Significantly different from the contusion only group, as determined by ANOVA plus Dunnett’s *t* test; p<0.05.

### Schwann and OEG cell grafts increase the number of spared/regenerated propriospinal neurons

**Figure 8:**
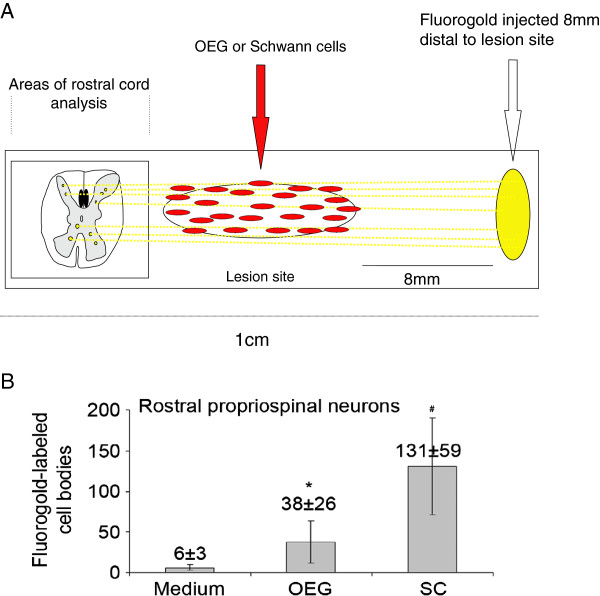
**Propriospinal neurons rostral to the lesion site were retrogradely labeled with Fluorogold injected caudal (8 mm) to the lesion site (A).** Significantly higher numbers of propriospinal neurons (n=10, p<0.05) were observed in the Schwann cell-transplanted group (#) and OEG-transplanted groups (*) relative to the medium-injected group **(B)**.

Fluorogold propriospinal neurons were counted from spinal cord segments rostral to the lesion/transplantation site (see schematic in Figure 8A). Propriospinal neurons that had reached the caudal injected Fluorogold (8 mm caudal from the caudal borders of the injury site) numbered 6±3 in the medium injected group 38±26 in the OEG transplanted group and 131±59 in the SCs transplanted group (see Figure 8B). As seen in Figure 8 there was a significant difference between the medium injected and OEG transplanted groups, but a significantly larger number of labeled propriospinal neurons was observed in the SCs group as compared to the medium injected group (p<0.03). There was no statistically significant difference between the OEG and SCs transplanted groups.

### Functional recovery: open field behavioral testing (BBB) and Ladder walk testing

**Figure 9:**
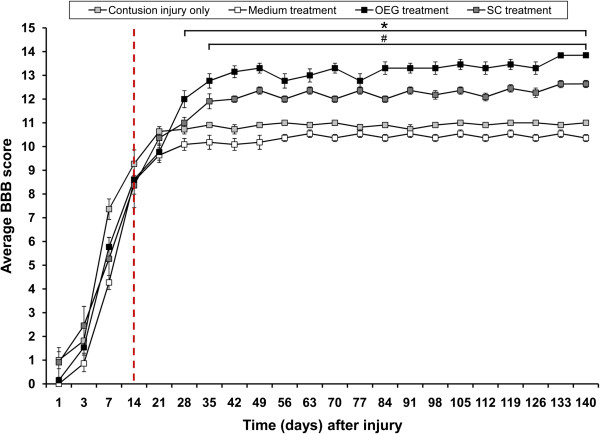
**Mean BBB scores over 4 months after injection of medium, Schwann cells and OEG plotted against contusion injury only.** The red dotted line depicts the transplant day. A statistically significant increase in BBB scores was seen in the Schwann cell group when compared to contusion only (#, p<0.05). A significant increase in BBB scores was also seen in the OEG group when compared to control injury only (*, p<0.05).

At two weeks after injury and again at two weeks after transplantation, rats in all groups had regained bladder function. Open field behavioral scoring indicated no statistically significant changes in the BBB scores in all groups before transplantation (Figure 9). One week following transplantation of OEG or SCs (i.e. 21 days post injury) these scores did not increase above control scores, with all groups scoring 10–11. At 2 weeks after treatment with cells or medium, control injury only rats scored a mean of 11, medium injected scored 10, OEG scored 12 and SCs scored 11 (Figure 9). Three weeks following injection of cells, the mean BBB scores for OEG injected rats were 13, SCs scored 12, medium injected scored 10 and injury only scored 11. Theses scores were consistent for all the following time points until day 133 and 144 when OEG scored a mean of 14, SCs scored 13, medium injected scored 11 and 10, and injury only scored 11 and 11 respectively (see Figure 9).

**Figure 10:**
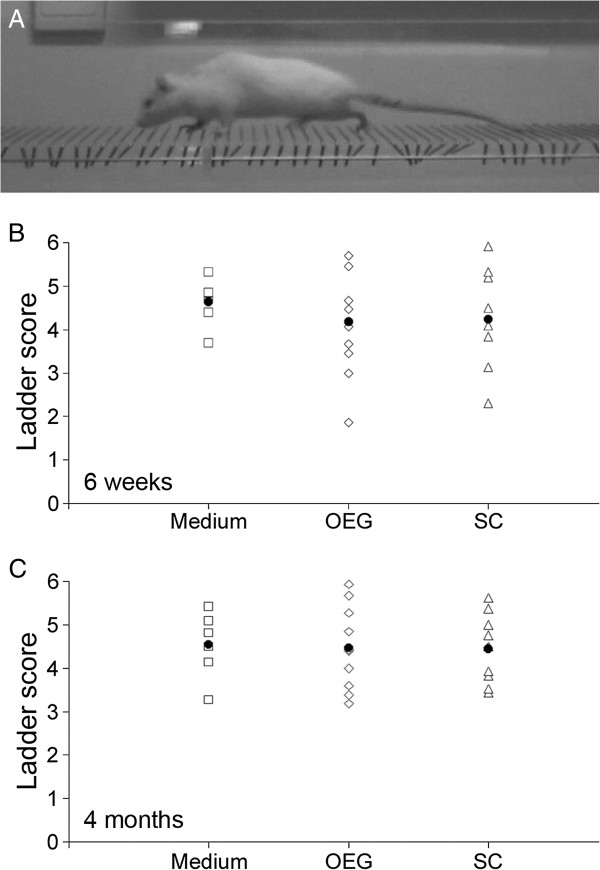
**Ladder walking behavioral testing. A**: representative photo of a rat traversing the horizontal ladder. **B**: ladder scores of treatment groups on horizontal ladder walking test 6 weeks after cell transplantation date. **C**: as for B at 4 months after cell transplantation date. At no stage were statistical differences between groups observed on the horizontal ladder test.

After one week of testing on the BBB score the rats were introduced to the ladder walk (see Figure 10A). At 6 weeks or 4 months after treatment, most rats scored between 3.5 and 5.5 (see Table 2) out of 6 on the horizontal ladder walking test (Figure 10B-C), with no significant differences observed between groups.

**Table 2:** **Scoring scale developed for the ladder rung walking test, modified from (Metz and Whishaw, [**[63]**])**

**Observation**	**Score**
Absent	0
Collapse	0
Deep slip then jump	1
Deep slip, unsuccessful or no paw replacement	1
Deep slip, paw replacement, slight slip	2
Step, unsuccessful paw replacement	2
Deep slip, successful paw replacement	3
Jump step	3
Step, paw replacement, slight slip	4
Step, good paw replacement	4
Slight slip, short stride	5
Slight slip, regular stride	5
Plantar paw placement, short stride	6
Plantar paw placement, regular stride	6

## Discussion

We have shown the capacity of purified, primary OEG and SCs to promote behavioral recovery, increase numbers of supraspinal axons, and increase tissue sparing when transplanted two weeks after a moderate spinal cord contusion injury. A lentiviral vector encoding DSRED-2 was used to transduce OEG and SCs – in this study it was shown to be an effective and stable transgene to label both cell types up to 4 months *in vivo* following transplantation. Viable transplanted OEG and SCs were found in similar numbers at 2 weeks, but after 4 months the OEG numbers were less than the number of SCs remaining. Histochemical tissue analysis showed a significant reduction in tissue loss in both the OEG and SCs groups 4 months post-transplantation. Further, dorsal white matter tracts appeared to be better protected in the transplant groups than in non-cellular transplanted controls (medium). Analysis of DSRED-2 labeled glia in the spinal cords showed a migration/dispersal difference between the two cell types. OEG were more evenly distributed in a rostro-caudal direction, and greater integration of OEG with host astrocytes was evident when compared to the tight interwoven grafts in the SCs transplanted rats. OEG transplanted rats displayed significantly lower levels of CS56 immunofluorescence within and around the lesion/transplant site when compared to the SCs group. Matrix deposition was similar between OEG and SCs groups, but laminin and collagen IV immunofluorescence showed a closer co-existence of these matrix molecules to SCs than to OEG. Axonal staining using neurofilament antibodies showed tighter bundles of axons within the SCs transplant zone than in OEG zones. Open field behavioral testing (BBB) showed significant differences between the transplanted groups and the controls up to 4 months after transplantation. BBB scores were consistently higher in the OEG group compared to control injury, medium and SCs injected rats. Additional behavioral testing using the ladder walk however, did not show any significant differences between experimental and control groups. Fluorogold retrograde tracing 8 mm distal to the injury/transplantation site revealed significant differences between control groups and OEG and SCs transplanted groups in supraspinal projecting axon populations. This provides strong evidence that a sub-acute transplant of SCs or OEG provide an environment in which promotion of sparing/regeneration of supraspinal axonal projections to the spinal cord are increased.

### SCs and OEG differ in their interaction with host spinal cord cells and matrix production

Evidence of statistically significant tissue-protective effects of OEG was observed 4 months after transplantation. At 2 weeks post-transplantation, tissue loss was still occurring. The tissue-protective effects of transplanted OEG or SCs arise from the secretion of factors from the cells such as BDNF, NGF, GDNF and NT4/5 [13,46-48] or matrix (laminin-1, collagen IV) and adhesion molecule (N-Cadherin) secretion [49-51]. These mechanisms of protection combined with tissue-rebuilding or new cell birth [52], are potentially acting together to improve anatomical functional outcomes.

Proliferation of donor OEG seems unlikely, as the labeling method employed in this study would result in all daughter cells also being labeled; the number of labeled cells at 4 months in the lesion site were fewer than when counted at 2 weeks. The fewer numbers of detectable OEG involves movement away from the site of injury (measurement area), or death (via apoptotic or anoikis mechanisms) [42,44]. In a small number of animals, OEG were observed several millimeters from the injection site which is similar to earlier studies which reported OEG migration at distances of 9 mm from the injection site [9,53,54]. In contrast to the OEG, SCs were not observed very far from the immediate lesion area, dispersing only up to a distance of 1.5 mm. A similar lack of dispersal has been reported out of peripheral nerve grafts inserted into the adult CNS [55,56]. This is likely due to interactions with host astrocytes and other endogenous non-neuronal cells [57].

The observed difference in the amount of intact tissue between the OEG and SCs transplanted groups indicates chemotropic effects of the OEG [58], similar to the chemotropic effects exerted by glia during development [59] that result in increased infiltration of various cells into the lesion site. OEG have the capacity to stimulate a different inflammatory response by the secretion of chemokines such as CX3CL1 [60], which is a known ligand for the receptor CX3CR1 found on microglia and dendritic cells [60,61] and affects the amount of tissue preservation/removal. OEG potentially are be more susceptible to death and subsequent phagocytosis by macrophages than SCs. OEG are known to secrete different factors and have unique antigenic profiles other than the ones mentioned above which make them more susceptible to immune attack [62].

OEG and SCs transplants have the properties to directly influence proliferation or differentiation of spinal cord progenitor cells/stem cells [52,63]. Glial transplants have the potential to differentiate these stem cells, like fibroblasts [64], into neurons or myelinating glia [64-67]. In our study we did not examine the direct OEG or SCs myelination potential or host myelination within the lesion site. Both cell types have the capacity to myelinate but differ in their effectiveness and mechanisms involved [75, Plant et al., -unpublished observations]. The proliferation of endogenous cells does occur in SCI [45]. These cells can include progenitor cells, endothelial and pericytes stimulated by factors such as epidermal growth factor and/or fibroblast growth factor-2 [68-70]. Proliferation seems highly plausible especially when OEG and SCs are known to produce similar levels of FGF-2 [71]. Two recent findings have indicated new cells play a role in SCI. New evidence from Goritz and colleagues [72] has indicated that pericytes play a role in the spinal cord injury response, whereby these cells actively migrate to sites of injury. OEG and SCs in our model are likely to have altered the number of these cells in the lesion site, thus providing a reparative outcome. In addition, evidence by Decimo and colleagues [73] found new adult stem cell /precursor cells that are resident in the meninges react to SCI and populate the injury site, thus providing a target for glial cell differentiation or activation.

In this study we provide evidence that following spinal cord contusion injury, endogenous repair processes including SCs migration were ongoing. Proliferation of ependymal cells in the walls of the central canal and from adjacent intact parts of spinal cord [45,52,65,74,75] is one route. Ependymal cells are known to migrate and populate the lesion site along with new blood vessels, astrocytes and fibroblasts. This seems likely to have contributed to the cellular trabeculae and scar tissue seen within our lesion cysts [45,75,76]. Host SCs from the dorsal roots or blood vessels also infiltrated the lesion site in our model, based on large numbers of p75-positive/DSRED-2 negative cells in all groups, as has previously been observed in the rat [14,45,55,77,78] and human [79] with the majority from the dorsal roots [32].

In a previous study from our laboratory [57], we indicated that molecules (including neurotrophins) released by Schwann cells promote the reactivity of astrocytes. Transplanted SCs stimulate astrocytes to be highly GFAP positive and surrounded by CS56 staining. This forms a dense glial scar around the lesion and prevents the entry of axons, and/or other host non-neuronal cells [57]. In this study we demonstrate in sub-acute 14 day glial transplants, OEG were associated with significantly lower CS56 expression and slightly higher collagen IV expression in the lesion and graft site compared to SCs. Since OEG are proficient in interacting with astrocytes *in vitro*[49,80], their release of extracellular matrix and cell adhesion molecules and their modulation of these factors may also account for the observed axonal infiltration into the glial scar [81]. Molecules such as Fibulin-3 and its ability to bind TIMP-3 may also play a part in the different interaction with host glia [26]. OEG isolated from the olfactory bulb show high levels of this protein when compared to SCs [26]. One recent report has also suggested an important role of MMP’s in cell migration and modulation of the ECM [82]. While both OEG and SCs appeared to be associated with a greater quantity of extracellular matrix molecules in the lesion site compared to the effect of medium injection alone, their profiles differed. The laminin was present in a complementary and non-overlapping pattern to DSRED-2 labeled SCs and OEG. SCs appeared to produce basement membrane, while OEG are capable of producing these molecules but not in the quantity seen in SCs grafted animals; rather, they may have induced other cells to produce extracellular matrix such as astrocytes or endothelial cells. This is not surprising given the role the OEG play in the olfactory system, where OEG form channels which stay present even after axonal degeneration in the olfactory bulb for axons to grow through and out to innervate areas of the olfactory bulb [83,84]. One mechanism for OEG reparative outcomes in the CNS is their close “cross-talking” with fibroblasts/meningeal cells [see [48,81] and ability to interact with these cells [9,85]. OEGs have a very close relationship with fibroblasts in the olfactory system, as do SCs in the peripheral nerve, but their role seems very different. SCs are potentiated to myelinate axons by fibroblasts and their associated matrix [86], whereas OEG interact in an axonal replacement/regenerating system without myelination [87]. In addition they have previously been shown play a role in fascicle-forming arrangements without fibroblasts [88-90].

### Olfactory glia and Schwann cells increase axonal sparing/regeneration in sub acute treated SCI

Our results show that transplantation of OEG or SCs at 2 weeks after contusion facilitated significant axonal sparing/regeneration comparable to those seen in transplants carried out 7 days after a contusion injury [33]. Significant increases in regenerated propriospinal fibers were also present in both graft groups, but numbers in SCs treated animals were significantly higher than after OEG transplantation. This indicates a more potent, local secretion of factors by SCs compared to OEG, but as yet no study has extensively analyzed these differences. In this study, an OEG or SCs transplantation following a contusion injury was associated with an increased amount of intact tissue at the lesion site. Immunohistochemistry for neurofilament-positive axons showed strongly immunoreactive profiles at the lesion site for both OEG and SCs transplanted groups after a 14 day delay of transplantation. Neurofilament positive axons in the SCs group were less linearly organized than those seen in the OEG group. This could be explained by the interaction with host astrocytes not allowing for the even distribution of the cells and the formation of a clear cellular boundary [91].

Retrograde tracing using Fluorogold revealed statistically significant increased numbers for supraspinal tracts after OEG or SCs transplantation. Supraspinal spinal tracts such as reticular, vestibular and hypothalamic were seen to respond similarly in both transplant groups. It should be noted that these supraspinal fibers were measured from 8 mm distal to the injury site, and so possible differences in fiber numbers between the transplanted cell groups at the lesion site itself cannot be discounted. Unlike the other spinal tracts, the raphe tract responded in a far more robust fashion in the OEG group. This can be via increased responsiveness to BDNF from the OEG directly or by the interaction with activated microglia which are known to secrete BDNF after ATP activation through the P2X4 receptor [92]. OEG differ to SCs in their levels of fractalkine secretion, and this can activate microglia to become more active and secrete different neurotrophic profiles to that initiated by SCs that do not secrete this protein at the same level. An important consideration, that is difficult to assess at the time of cell delivery, is the stage of cellular differentiation of transplanted glia after a period of time *in vivo.* SCs will differentiate into myelin forming cells quite quickly thus reducing their regenerative/autocrine secretory products [93-95] whereas OEG stay in an undifferentiated [p75-positive) non-myelin forming state for a longer periods (Plant – unpublished observations) or may not myelinate at all [33].

### Functional recovery is improved after sub acute transplantation of OEG and SCs

Significant behavioral differences were observed between the control injury and medium injected rats, and both OEG and SCs grafted groups. Improvements, which are normally observed in studies similar to that undertaken here, may result from compensatory effects occurring within reflex-based segmental cord systems [96]. OEG transplantation into a completely transected spinal cord has elicited improved hindlimb function [9,10,97] as seen here. One possibility that could increase locomotion is increased noradrenergic and serotonergic varicosities apposing the motor associated cholinergic neurons [98]. OEG treated rats showed significant numbers of raphe projecting axons reaching at least 8 mm distal from the injury site and these could stimulate such a behavioral response. This however does not explain the SCs grafted rats which may use an alternate but effective pathway of locomotion. SCs induced recovery could be partially explained by a graft relay system developed through regeneration of propriospinal axons regeneration as seen in our results here. Only small numbers of spared fibers (<5%) are required to facilitate compensatory return of function via segmental circuits [99]. Phasic behavioral recovery in the SCs group, in particular 49 days until the end of the experiment, may be due to phasic secretion of neurotrophins which may not be delivered at the same level via the transplantation of OEG.

## Conclusions

Glial cells transplanted at 14 days post injury may experience a less accommodating lesion site than when grafted 1 week after injury, which may explain a reduced glial survival in particular for OEG than seen in earlier transplant studies. At 14 days post SCI proteoglycan deposition at the lesion site is present and highly inhibitory [100,101] but was shown to be reduced in the OEG transplanted groups providing a positive milieu for the sparing/regeneration of supraspinal axons (serotonergic positive fibers). This evidence is strongly backed by our use of fluorogold tracing, but was not supported by immunohistochemistry for 5-HT [98] (which labels serotonergic axons innervating the spinal cord). Supplementation of glial transplants with neurotrophins may improve cell survivability [13] and in combination with removal of the glial scar by the use of chondroitinase could potentially improve axonal regeneration in this 14 day delayed glial transplant model [102,103]. Clearly the delay between injury and treatment can have a strong bearing on the experimental outcome but also the success of the glial type to improve tissue and behavioral outcomes, modify the lesion site and improve sparing/regeneration of axonal populations as shown here. From the present study it would appear that a 14 day delay in glial transplantation is feasible and beneficial both anatomically and behaviorally after a contusive spinal cord injury but the mechanisms of repair of the two glial types are different. We have examined two time points (2 weeks and 4 months) using multiple behavioral and anatomical analysis, but results can only give an indication of efficacy. Further physiological, behavioral and anatomical correlative studies using these glial cells at 14 days post injury time point would provide valuable evidence of cell potential and reproducibility for future CNS repair applications.

## Methods

### Animals

Adult (180-200 g) female Fischer 344 rats (Animal Resource Centre, Murdoch, Western Australia) were housed in groups of three under controlled conditions of temperature and humidity, with free access to food and water. All procedures were approved by the Animal Ethics Committee of The University of Western Australia (approval number 99/008/E42). Fourteen days after injury, previously described injection procedures [8] were used to transplant OEG (n=6 short term 2 weeks, and n=11 long term 4 months) or SCs (n=6 short term 2 weeks and n=11 long term 4 months) into the lesion site controls received an injection of cell culture medium (n=6 short term 2 weeks and n=11 long term 4 months) or injury alone (n=6 short term 2 weeks and n=11 long term 4 months). Six additional rats were perfused 2 weeks after contusion without a transplant, to examine the lesion milieu into which transplanted cells would have been introduced. Total rats used n=74. All rats receiving a transplant and/or medium were perfused at 2 weeks or 4 months after transplantation. The numbers of animals were final numbers analyzed. In the long term groups 3 animals died and in the short term groups 6 rats.

### Olfactory ensheathing glia and Schwann cell culture and purification

OEG were prepared from the olfactory bulbs of adult female Fischer 344 rats [8,13,33]. In brief, the bulbs were extirpated and the olfactory nerve layer dissected. Tissue was mechanically dissociated and treated with trypsin 0.5% w/v (Worthington Biochemical Corporation, Lakewood, NJ) in Hank’s Balanced Saline Solution (Sigma) for 60 minutes at 37°C in a CO_2_-free incubator. Tissue was pelleted by centrifugation at 1500 rpm for 5 minutes, resuspended in DF10S medium (1:1 DMEM/F-12 -Sigma), 10% (v/v) fetal calf serum - Hunter Antisera) supplemented with bovine pituitary extract (20 μg/mL Gibco) and forskolin (2 μM Sigma), then plated on poly-l-lysine-coated culture dishes (Corning, Acton, MA) for one week. OEG were purified by immunoaffinity to an antibody to the p75 receptor (192-IgG) [13,33].

SCs were isolated from adult female Fischer 344 rats [19,33]. Briefly, the sciatic nerves were dissected, placed in 35 mm uncoated tissue culture dishes in D10S medium consisting of DMEM (Sigma St Louis, MO) with 10% fetal calf serum (Hunter Antisera Jesmond, NSW, Australia) and cut into 1-2 mm^3^ pieces. Each week, nerve segments were transferred to a new culture dish, leaving behind fibroblasts that had migrated out of the explants. After three weeks of fibroblast depletion, explants were enzymatically and mechanically dissociated and transferred to new dishes for culture in the presence of bovine pituitary extract (20 μg/mL Gibco, Carlsbad, CA) and forskolin (2 μM Sigma) [22].

### Lentiviral labelling of glia

Lentiviral vectors encoding the gene for DSRED-2 were made according to methods described by [104]. Cultured OEG or SCs were labeled by adding the vector (using a multiplicity of infection of 100 [12] to the culture medium for 16 hours, followed by 2 washes in culture medium and fresh media for growth.

### Spinal cord injury and post-operative care

Anesthesia was induced with 5% (v/v) halothane (in 60:40 O_2_:N_2_O) then maintained at 2-3%. The skin and muscle layers were cut rostro-caudally to expose the vertebral column, and a T10 laminectomy performed [8] Using an New York University (NYU) impactor, a moderate contusion injury was induced by dropping a 10 g weight from a 12.5 mm height [99,105] Gel-Foam (Pharmacia & Upjohn, Kalamazoo, MI) was used to stop bleeding in some animals. Animals were partially suspended from clamps to the dorsal processes of T8 and T11/12, to circumvent diaphragm-induced dorso-ventral movement of the spinal column. Immediately after the injury, the animal was removed from the clamps and the muscles sutured in layers with Vicryl (Johnson & Johnson, New Brunswick, NJ). Skin was closed with Michel suture clips (Fine Science Tools Inc., North Vancouver, BC, Canada). Immediately after surgery, each animal was injected with penicillin (Benacillin intramuscular), analgesic (Temgesic buprenorphine sub-cutaneous 0.1 mg/kg) and 2-4 mL 0.9% saline (sub-cutaneous). Animal cages were placed partially onto a heating pad for 24 hours, to assist with maintenance of body temperature. Antibiotics were continued on alternate days, with analgesic twice daily and saline once daily for one week. Manual bladder expression was performed twice daily for two to three weeks, by which time all animals had recovered bladder tone.

### Cell transplantation

Cells were transplanted after no more than 2 weeks *in vitro*[8]. Fourteen days post-contusion, adult female rats were anesthetized and the spinal cord exposed as described above. Either 5 μL of a cell suspension containing 5 × 10^5^ OEG or SCs in culture medium, or medium alone, (1:1 DMEM/F-12 Sigma) was injected into the center of the lesion over a period of 3 minutes using a 5 μL syringe (Hamilton, Reno, NV) mounted in a stereotaxic frame. The needle was withdrawn 3 minutes later to encourage cell integration into the injection site. Wounds were sutured and post-operative care was administered as described above.

### Behavioral testing

All testing of the BBB and ladder walk were carried out by observers blinded to the treatments. BBB testing [99] was performed on each animal on the day following contusion, to confirm the successful lesion (a score of 0). The BBB test was repeated on day 3, day 7 and day 14 after contusion thereafter every week after cell transplantation. 3 weeks post-injury, animals were consistently scoring BBB results of 10–11. A ladder rung walking test modified from [106] was introduced 1 week after injury and measured every week for 4 months. The ladder rung walking test provided 6 points (Table 2) for scoring, thus allowing a greater level of sensitivity than the two BBB points by which they were separated.

The ladder rung walking test [106] apparatus was 1.5 m in length and consisted of two clear Perspex sidewalls and a floor with removable metal rungs spaced 1 cm apart. Prior to surgery, rats were trained to walk along the ladder to reach their home cage. From 1 week after injury, trials were performed weekly and video recorded. The animals had 3 runs per day when tested. The video camera was positioned to capture the central portion of the ladder, approximately 30 cm in length, allowing analysis of approximately 8 steps of each hind limb. Each hind limb step in each trial was later scored (Table 2) the scores were averaged to give a single value, per rat, per trial.

### Fluorogold labeling

Ten days prior to perfusion (4 months post-transplantation of cells), 11 rats from each of the long term (4 month) groups were anesthetized as above and subjected to a T12 laminectomy. While held in a stereotaxic frame, Fluorogold (1 μL Fluorochrome Inc., Denver, CO) was injected into the spinal cord at 4 points (0.25 μL each), 2 segments caudal (8 mm) to the contusion site. All injections were performed by the same individual, to maximize comparability between animals this individual was also blinded as to which groups had previously been injected with OEG, SCs or control cells. Muscles were sutured in layers and the skin closed with Michel suture clips (Fine Science Tools Inc., North Vancouver, BC, Canada). Animals were treated with the post-operative care regime detailed above.

### Tissue processing

At 2 weeks or 4 months after Schwann cell or OEG transplantation, rats were terminally anesthetized with Nembutal (intraperitoneal sodium pentobarbital Merial Australia, Parramatta, NSW, Australia) and perfused intracardially with approximately 150 mL of heparinized phosphate-buffered saline (Heparin 1 U/mL David Bull Laboratories, Melbourne, VIC, Australia) followed by approximately 150 mL 4% buffered paraformaldehyde solution. The spinal cord and brain were immediately removed and post-fixed in buffered paraformaldehyde overnight at 4°C. The following day, paraformaldehyde was replaced with 0.1 M phosphate buffer. Prior to tissue sectioning, each spinal cord or brain was placed into sucrose (30% w/v in phosphate buffer) overnight and embedded in gelatin (10% w/v in phosphate buffer Difco, Franklin Lakes, NJ). The gelatin block was fixed by immersion in buffered paraformaldehyde for 2 hours and placed into buffered sucrose solution overnight. Tissue was sectioned (spinal cord at 40 μm brain at 50 μm) using a freezing sledge microtome and placed into phosphate buffer containing 0.05% w/v sodium azide.

### Tissue analysis - Quantification

Numbers of animals per group were 8–12 and based on: (i) a prior power analysis study to ascertain experimental numbers in animal models of spinal cord injuries, in order to appropriately assess statistical differences, and (ii) previous publications from the laboratory. All rats were tested with BBB, gridwalk, fluorogold traced, immunohistochemistry, and tissue analysis. These were randomly allocated to the groups without the knowledge of the surgeon or behaviorist. Animals were taken from the naïve cages and re-housed into post-surgery cages and numbered. This number was then combined by an independent person to identify the treatment. At every level the person was blinded to the treatment.

The perfusions were performed on the same day for all groups with the same fixative batch. The immunohistochemistry was also carried out between each of the groups reducing differences in staining between days and groups. The analysis was performed by a trained viewer who was blinded to the groups.

### Tissue volume estimation

One in six sections from each spinal cord was mounted onto gelatin-coated microscope slides and stained with gold chloride solution for myelin followed by cresyl violet for Nissl substance [107]. Slides were dehydrated through an ethanol/toluene series and mounted in DPX (Chem Supply, Gillman, Australia). Sections were visualized using a Leica DM RBE microscope and digitally photographed. Image Pro Plus (Media Cybernetics, Silver Spring, MD) was used to measure the tissue area of a 4.5 mm section of spinal cord with the lesion center at its midpoint.

Tissue cavitation was measured in all animals to gain an estimate of the effect of the transplanted cells upon the preservation of tissue at the lesion site the volume of tissue remaining at the injury site was measured in a similar manner to that described previously [8,14,108] The combined area of any cysts was calculated and subtracted from the total tissue area of the section, to give a value for the total tissue remaining. Areas showing signs of degeneration, such as microcysts and loss of neuronal profiles, were subtracted from the values for total tissue remaining to obtain a measure of intact tissue. The values for the centre four sections of each spinal cord were averaged to give a single semi-quantitative value of remaining tissue volume value for each rat. Values from four equivalent sections of an uninjured spinal cord were used to compute the tissue remaining as a proportion of uninjured spinal cord.

### Immunohistochemistry

Series of one in six tissue sections (in 2 week or 4 month time points) were immunostained free-floating with mouse monoclonal or rabbit polyclonal antibodies. Sections were incubated overnight at 4°C in primary antibody diluted in phosphate buffer containing 10% normal goat serum and 0.2% (v/v) Triton X-100. Mouse monoclonal antibodies used were: i) anti-chondroitin sulfate proteoglycans (CS-56 1:50 Sigma) to identify proteoglycan deposition at the lesion site, ii) low affinity nerve growth factor receptor p75 (supernatant 192-IgG) to stain for OEG and Schwann cells (both transplanted and endogenous) and iii) anti medium/high neurofilaments RT-97 (supernatant Developmental Hybridoma Bank) to stain for spared and regenerated axons. Rabbit polyclonal antibodies were used for: i) p75 (1:200 Promega, Madison, WI), Glial fibrillary acidic protein (GFAP) (1:500 DakoCytomation, Denmark) to stain for astrocytes, unmyelinated Schwann cells and olfactory glia, ii) anti-collagen IV (1:200 Rockland Inc., Gilbertsville, PA) to ascertain the distribution of basal lamina and blood vessels and iii) anti-laminin-1 (1:200 Sigma) for basal lamina and blood vessels.

The following day, sections were washed three times with phosphate buffer and incubated with secondary antibody (diluted in the same solution as for primary antibodies) for 30 minutes at room temperature. Sections were washed three times with phosphate buffer, mounted onto gelatin-coated glass microscope slides, air dried and then coverslipped in a glycerol-based mounting medium.

### Semi-quantitative analysis of proteoglycan, matrix and cell survival *in vivo*

One in 6 series of sections at 4 months post transplantation (and controls) were analyzed. Low magnification photomicrographs (10× objective) were taken using an IX70 Olympus microscope. Pixel fluorescence of immunostaining for CS-56, laminin and collagen IV was measured at rostral, middle and caudal locations of the contusion/injury sites and the average pixel fluorescence minus background readings of the three areas compared between medium control and OEG or SCs groups [37,57]. In addition, one in 4 series of sections were analyzed for the presence of DSRED-2 labeled cells in the 2 week and 4 month survival groups. Pixel fluorescence was then compared between the 2 survival points to estimate survival of OEG and SCs at 4 months expressed as a percentage of those present at 2 weeks.

### Fluorogold photography and counts

A 1 in 6 series of brain sections were mounted onto gelatin-coated glass microscope slides, air-dried and mounted in a glycerol-based mounting medium. Sections were photographed with a Hitachi HV-C20M digital camera as a large tiled image using a fluorescence microscope (Leica DM RBE) with a motorized stage controlled with Image Pro Plus (5.1) software (Media Cybernetics). The optical dissector method was used to photograph and count fluorogold-labeled neurons, in order to avoid inaccuracies due to double counting [109] Photographs were taken in the centre of the z-axis of each section. Those cell bodies in focus were identified, then tagged and counted using Image Pro Plus. Seven areas were analyzed: the reticular formation, vestibular nucleus, trigeminal and dorsal column nuclei, raphe nucleus, red nucleus, hypothalamus and motor cortex.

To quantify sparing/regeneration of descending propriospinal neurons, the labeled cell bodies were counted using an identical method in the spinal cord region rostral to the lesion site. Six spinal cord sections (1 cm in length) from each animal were analyzed.

### Statistical analysis

The methods of statistical analysis were chosen that reflected the animal numbers used in this study. For Fluorogold labeling of supraspinal projecting nuclei in the brain, experimental groups were compared using a one-way analysis of variance (ANOVA) followed by the Dunnetts methods of multiple comparisons versus a single control group (injury only) and also the Tukey test for multiple comparison procedures between all experimental and control groups. Fluorogold analysis of propriospinal projections into the distal spinal cord were analyzed using a one way analysis of variance (ANOVA). This was also the case for the tissue sparing and proteoglycan and cell survival analysis. In the analysis of the BBB scores, groups were compared using the non parametric Kruskal Wallace ANOVA on ranks. This was followed by Dunnetts method of multiple comparisons versus a single control group.

## Abbreviations

OEG: Olfactory ensheathing glia; SCs: Schwann cells; BBB: Basso, Beattie and Bresnahan; ANOVA: One way analysis of variance; SCI: Spinal cord injury; GFP: Green fluorescent protein; GFAP: Glial fibrillary acidic protein; BDNF: Brain derived neurotrophic factor; NGF: Nerve growth factor; GDNF: Glial derived neurotrophic factor; NT4/5: Neurotrophin 4/5; DMEM: Dulbecco’s modified eagle’s medium; NYU: New York University.

## Competing interests

The authors declare that they have no competing interests.

## Authors’ contributions

HB contributed to the acquisition of data, analysis and interpretation. CDP contributed to the analysis of data, drafting of manuscript and revising for intellectual content. ARH contributed to the analysis of data, drafting of manuscript and intellectual content. GWP conceived of the study and design, acquisition of data, analysis and drafted the manuscript. All authors have read and approved the manuscript.

## Supplementary Material

Additional file 1: Table S1 DSRED-2 Fluorescence Counts in OEG Transplanted and SC Transplanted groups. Click here for file
