# Supplementary material for: Tissue sparing, behavioral recovery, supraspinal axonal sparing/regeneration following sub-acute glial transplantation in a model of spinal cord contusion
Source: BMC Neurosci. 2013 Sep 27;14:106. doi: 10.1186/1471-2202-14-106 (PMC3849889; doi:10.1186/1471-2202-14-106)
Supplement: Additional file 1: Table S1 — DSRED-2 Fluorescence Counts in OEG Transplanted and SC Transplanted groups. [file 1471-2202-14-106-S1.docx]

**Additional file 1: Table S1. DSRED-2 Fluorescence Counts in OEG Transplanted and SC Transplanted groups**

|  |  | *Short term treatment (2 weeks)* | | | | | |  | *Long term treatment (4 months)* | | | | | |
| --- | --- | --- | --- | --- | --- | --- | --- | --- | --- | --- | --- | --- | --- | --- |
|  |  | *Rostral* | *Mean % of total* | *Center* | *Mean % of total* | *Caudal* | *Mean % of total* |  | *Rostral* | *Mean % of total* | *Center* | *Mean % of total* | *Caudal* | *Mean % of total* |
| *OEG treatment group* |  | 70.17±28.65 | 29% | 85.67±34.97 | 36% | 83.67±34.16 | 35% |  | 22.17±9.05 | 36% | 21.00±8.57 | 34% | 19.17±7.82 | 31% |
| *SC treatment group* |  | 91.83±37.49 | 34% | 100.67±41.10 | 37% | 79.17±32.32 | 29% |  | 33.67±13.74 | 37% | 28.50±11.64 | 31% | 28.33±11.57 | 31% |

Data are presented as means ± SEM.

Alternative presentation:

|  | *OEG treatment group* | *SC treatment group* |
| --- | --- | --- |
| ***2 weeks post-treatment*** |  |  |
| *Rostral* | 70.17±28.65 | 91.83±37.49 |
| *Mean % of total* | 29% | 34% |
|  |  |  |
| *Center* | 85.67±34.97 | 100.67±41.10 |
| *Mean % of total* | 36% | 37% |
|  |  |  |
| *Caudal* | 83.67±34.16 | 79.17±32.32 |
| *Mean % of total* | 35% | 29% |
| ***4 months post-treatment*** |  |  |
| *Rostral* | 22.17±9.05 | 33.67±13.74 |
| *Mean % of total* | 36% | 37% |
|  |  |  |
| *Center* | 21.00±8.57 | 28.50±11.64 |
| *Mean % of total* | 34% | 31% |
|  |  |  |
| *Caudal* | 19.17±7.82 | 28.33±11.57 |
| *Mean % of total* | 31% | 31% |
| ***% reduction in mean fluorescence  at 4 months vs. 2 weeks*** |  |  |
| *Rostral* | 68% | 63% |
|  |  |  |
| *Center* | 75% | 72% |
|  |  |  |
| *Caudal* | 77% | 67% |
|  |  |  |
| *TOTAL* | *74%* | *67%* |
